# Sustained innovation: Monash Health Wellness and Recovery Centre, treatment of eating disorders

**DOI:** 10.1186/2050-2974-3-S1-O12

**Published:** 2015-11-23

**Authors:** Lawrence Harvey

**Affiliations:** 1 Monash Health, Australia

## Aim

Eating Disorders are serious and potentially life threatening conditions with major physical and psychological sequela. Research indicates that the complex nature of Eating Disorders requires specialised services to effectively manage and treat clients.

## Design

The Mental Health Program led an organisational wide review of the management of Eating Disorder clients at Monash Health. This included collating and analysing information from expert working parties, focus groups, and forums, analysis of data, as well as surveys that were distributed to carers, consumers, Monash Health employees, and external agencies working with clients with Eating Disorders. The findings indicated a number of issues with the current service, including multiple referral pathways, poor interface between primary, acute, and mental health, no shared care approaches to treatment, and lack of consumers and carers involvement in treatment.

## Method

The Monash Health Butterfly Eating Disorders Day Program was developed in 2007 as a joint 3 year pilot project between The Butterfly Foundation and Monash Health. The day program offers highly specialised treatment to individuals who require more intensive support than can be achieved in outpatient care alone. Following its successful implementation, the day program received recurrent funding from DOH. The findings of the Eating Disorder service review, and the success of the day program supported the need for further specialist Eating Disorder services within Monash Health. The Wellness and Recovery Centre (WRC) was developed in 2010, and represented a consolidation and redevelopment of Eating Disorder services. This included the day program, and the development of a specialised outpatient Eating Disorder treatment service utilising Cognitive Behavioural Therapy – Enhanced (CBT-E) as the first line treatment. An adult Eating Disorder inpatient unit was established to provide comprehensive and integrated medical, psychiatric, and nutritional assessment and treatment. An experienced and multidisciplinary clinical team was established across all of WRC to provide the specialised Eating Disorder treatment.

## Results

Results indicated significant improvement in clinical symptoms (including BMI, BAI, BDI, HONOS, SOC, EDQLS, EDI, EDEQ), and system outcome measures (e.g. reduction in ED presentations).

## Conclusion

Organisational review and redevelopment of a specialised Eating Disorder treatment service has led to improved clinical and system related outcomes.

